# The effect of bovine viral diarrhea virus (BVDV) strains on bovine monocyte-derived dendritic cells (Mo-DC) phenotype and capacity to produce BVDV

**DOI:** 10.1186/1743-422X-11-44

**Published:** 2014-03-07

**Authors:** Mrigendra KS Rajput, Mahmoud F Darweesh, Kaci Park, Lyle J Braun, Waithaka Mwangi, Alan J Young, Christopher CL Chase

**Affiliations:** 1 Department of Veterinary and Biomedical Sciences, SDSU, Brookings, SD 57007, USA; 2 Department of Veterinary Pathobiology, Texas A&M University, College Station, TX 77843, USA

**Keywords:** Monocytes, Monocyte-derived dendritic cells, Bovine viral diarrhea virus, Cytopathic BVDV, Non-cytopathic BVDV, Immunosuppression

## Abstract

**Background:**

Dendritic cells (DC) are important antigen presentation cells that monitor, process, and present antigen to T cells. Viruses that infect DC can have a devastating impact on the immune system. In this study, the ability of bovine viral diarrhea virus (BVDV) to replicate and produce infectious virus in monocyte-derived dendritic cells (Mo-DC) and monocytes was studied. The study also examined the effect of BVDV infection on Mo-DC expression of cell surface markers, including MHCI, MHCII, and CD86, which are critical for DC function in immune response.

**Methods:**

Peripheral blood mononuclear cells (PBMCs) were isolated from bovine blood through gradient centrifugation. The adherent monocytes were isolated from PBMCs and differentiated into Mo-DC using bovine recombinant interleukin-4 (IL-4) and granulocyte-macrophage colony-stimulating factor (GMCSF). To determine the effect of BVDV on Mo-DC, four strains of BVDV were used including the severe acute non-cytopathic (ncp) BVDV2a-1373; moderate acute ncp BVDV2a 28508-5; and a homologous virus pair [i.e., cytopathic (cp) BVDV1b TGAC and ncp BVDV1b TGAN]. The Cooper strain of bovine herpesvirus 1 (BHV1) was used as the control virus. Mo-DC were infected with one of the BVDV strains or BHV-1 and were subsequently examined for virus replication, virus production, and the effect on MHCI, MHCII, and CD86 expression.

**Results:**

The ability of monocytes to produce infectious virus reduced as monocytes differentiated to Mo-DC, and was completely lost at 120 hours of maturation. Interestingly, viral RNA increased throughout the course of infection in Mo-DC, and the viral non-structural (NS5A) and envelope (E2) proteins were expressed. The ncp strains of BVDV down-regulated while cp strain up-regulated the expression of the MHCI, MHCII, and CD86 on Mo-DC.

**Conclusions:**

The study revealed that the ability of Mo-DC to produce infectious virus was reduced with its differentiation from monocytes to Mo-DC. The inability to produce infectious virus may be due to a hindrance of virus packaging or release mechanisms. Additionally, the study demonstrated that ncp BVDV down-regulated and cp BVDV up-regulated the expression of Mo-DC cell surface markers MHCI, MHCII, and CD86, which are important in the mounting of immune responses.

## Background

Bovine viral diarrhea virus (BVDV) is an important disease of the cattle industry in the USA and worldwide. The virus belongs to the family *Flaviviridae* and is a single-stranded, positive-sense RNA virus with a genome of approximately 12.5 kb [1]. BVDV can further be classified on the basis of genotype and biotypes. Specifically, genotypes are divided into Type 1 (BVDV1) or Type 2 (BVDV2), and are distinguishable using monoclonal antibodies [2], while BVDV biotypes are classified as either cytopathic (cp) or non-cytopathic (ncp), based on the effect the virus has on cell culture [3]. Likewise, BVDV infection in cattle has multiple variations. Infection of the bovine fetus with ncp BVDV during the first trimester often results in persistently infected (PI) calves that are immunotolerant to BVDV, and thus remain a source of infection to other animals. Additionally, superinfection of PI animals with an antigenically homologous cp strain of BVDV typically results in fatal mucosal disease [4]. In the acutely infected animal, initial infection and replication of BVDV occurs in the oronasal mucosa and oropharyngeal lymphoid tissue [5], and the subsequent systemic spread occurs through lymphatic and blood circulation systems [6]. The BVDV virus can be detected in the blood approximately 4 to 8 days after initial exposure, and the buffy coat sample comprises the white blood cells (WBCs) present in peripheral blood, typically contain more virus than serum [7]. The buffy coat contains various antigen presenting cells including monocytes and dendritic cells. The DC actively provides surveillance for antigen and presents it to T cells after processing. As such, the infected DC may play an important role in BVDV dissemination in body. The infected DC may have altered surface marker expression that interferes with mounting an effective immune response. In this study, Mo-DC were used as an *in vitro* model of DC. The ability of BVDV to replicate and produce infectious virus in monocytes and Mo-DC was investigated along with the effect of BVDV infection on MHCI, MHCII, or CD86 expression. Four strains of BVDV were used in this study including the severe acute ncp BVDV2a 1373 strain, the moderate acute ncp BVDV2a 28508-5 strain, and a virus pair (cp BVDV1b TGAC and ncp BVDV1b TGAN) recovered from an animal that died of mucosal disease.

## Results

### Characterization of Mo-DC

**Figure 1:**
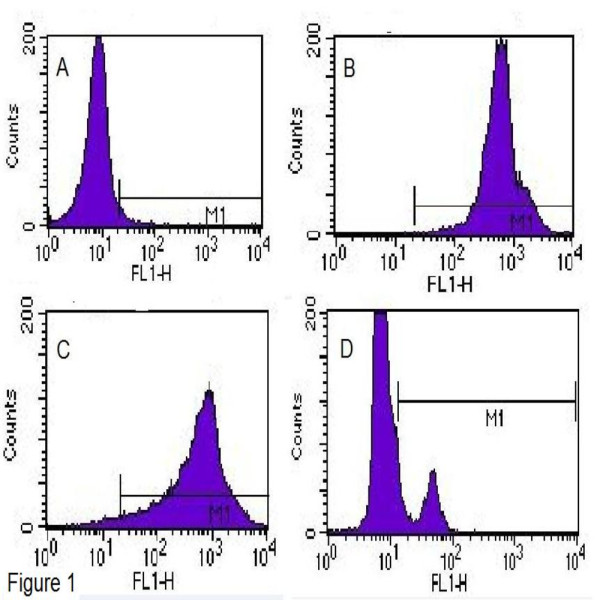
**Phenotype of adherent bovine monocytes.** The adherent monocytes were stained with MHCI, MHCII or CD14 primary antibodies followed by FITC-labeled secondary antibody. The experiments were repeated three times and mean expressions were calculated. **A)** Mean of secondary antibody control showing 2.03 ± 0.7% stained cells; **B)** Cells showing mean MHCI expression as 96.62 ± 0.5%; **C)** Cells showing mean MHCII expression as 80.58 ± 19.7%; and **D)** Cells showing mean CD14 expression as16.54 ± 1.5%.

**Figure 2:**
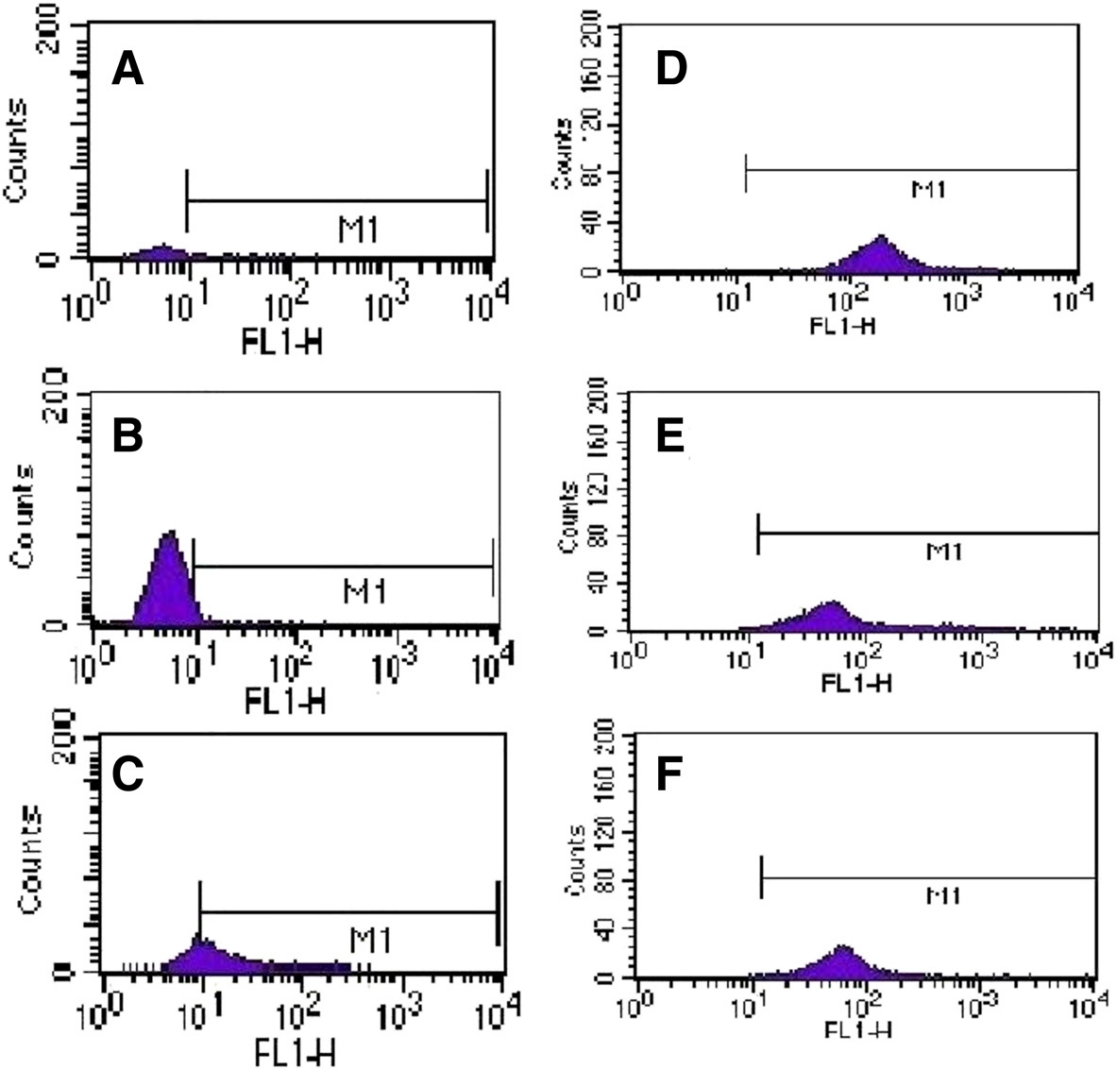
**Phenotype of Bovine Monocyte-Derived Dendritic Cell (Mo-DC).** The Mo-DC at seven days of differentiation were stained for CD14, CD21, CD205, MHCI, MHCII or CD86. The experiments were repeated three times and mean expressions were calculated. **A)** Cells showing mean CD14 expression as 1.025 ± 0.45%; **B)** Cells showing mean CD21 expression as 3.42 ± 0.19%; **C**) Cells showing mean CD205 expression as 55.97 ± 45.48%; **D)** Cells showing mean MHCI expression as 98.58 ± 0.34%; **E)** Cells showing mean MHCII expression as 94.1 ± 2.81%; and **F)** Cells showing mean CD86 expression as 77.83 ± 17.83%.

**Figure 3:**
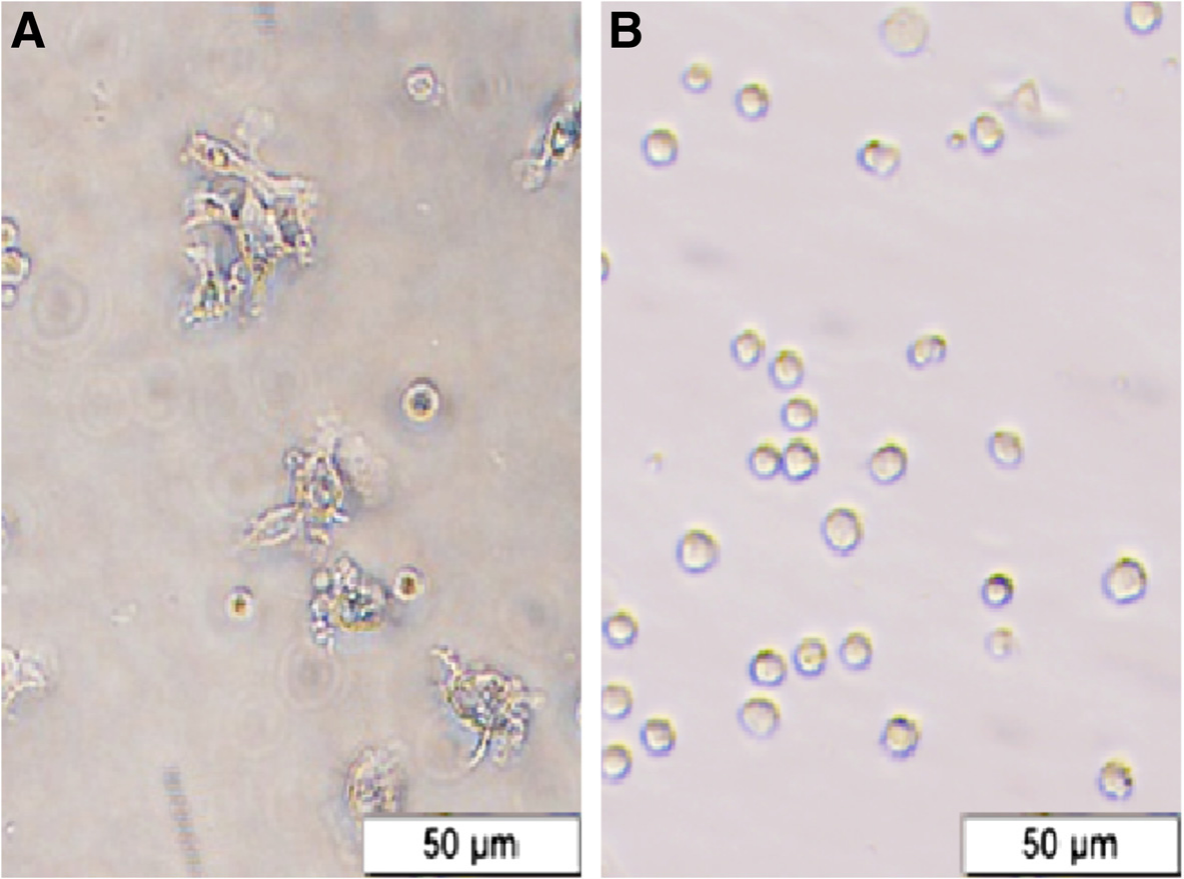
**Morphology of bovine monocyte derived dendritic cell. A)** The Mo-DC at seven days of differentiation with bovine recombinant IL-4 and GMCSF; **B)** Freshly collected monocytes.

Freshly collected monocytes were positive for MHCI (96.62 ± 0.50%), MHCII (80.58 ± 19.69%), and CD14 (16.54 ± 1.49%) (Figure 1). After 7 days of differentiation from monocytes to Mo-DC, the Mo-DC were positive for MHCI (98.58 ± 0.34%), MHCII (94.1 ± 2.81%), CD205 (55.97 ± 45.48%), and CD86 (77.83 ± 17.83%), and were negative for CD21 (3.42 ± 0.19%) and CD14 (1.025 ± 0.45) (Figure 2). During the 7 days of differentiation, the MHCI expressing cells increased from 96.62 ± 0.50% to 98.58 ± 0.34% (i.e., about 2%) and MHCII expressing cells increased from 80.58 ± 19.69% to 94.1 ± 2.81% (i.e., roughly 17%), while CD14 expressing cells were significantly (*P* < 0.05) reduced from 16.54 ± 1.49% to 1.025 ± 0.45 (i.e., about 94%). Morphologically, Mo-DC increased 4-5 times in size and developed long dendrites (Figure 3).

### BVDV do not affect the Mo-DC viability

**Figure 4:**
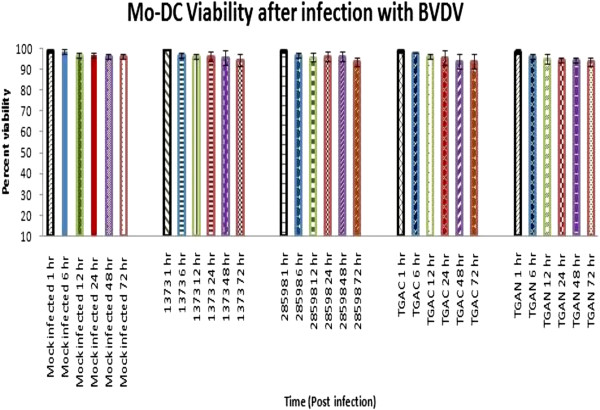
**Mo-DC viability after BVDV infection.** Mo-DC were infected with cp BVDV1b TGAC, ncp BVDV1b TGAN, ncp BVDV2a 28508-5, or ncp BVDV2a 1373 with a 6 MOI. The viability of Mo-DC was measured by trypan blue exclusion assay. The each experiment was repeated three times and mean percentages of viable cells were calculated from 1, 6, 12, 24, 48, and 72 hour(s) post-infection.

To determine the effect of BVDV infection on Mo-DC viability, Mo-DC were infected with cp BVDV1b TGAC, ncp BVDV1b TGAN, ncp BVDV2b 1373, or ncp BVDV2b 28508-5 strains of BVDV at a multiplicity of infection (MOI) of 6. Infected and mock-infected control Mo-DC were collected at 1, 6, 12, 24, 48, and 72 hour(s) post-infection. The viability of Mo-DC was determined via trypan blue exclusion assay, as described by Strober [8]. Results revealed that Mo-DC viability was not altered significantly (p > 0.05) during the course of infection, regardless of BVDV strain (Figure 4).

### Mo-DC do not produce infectious BVDV

**Figure 5:**
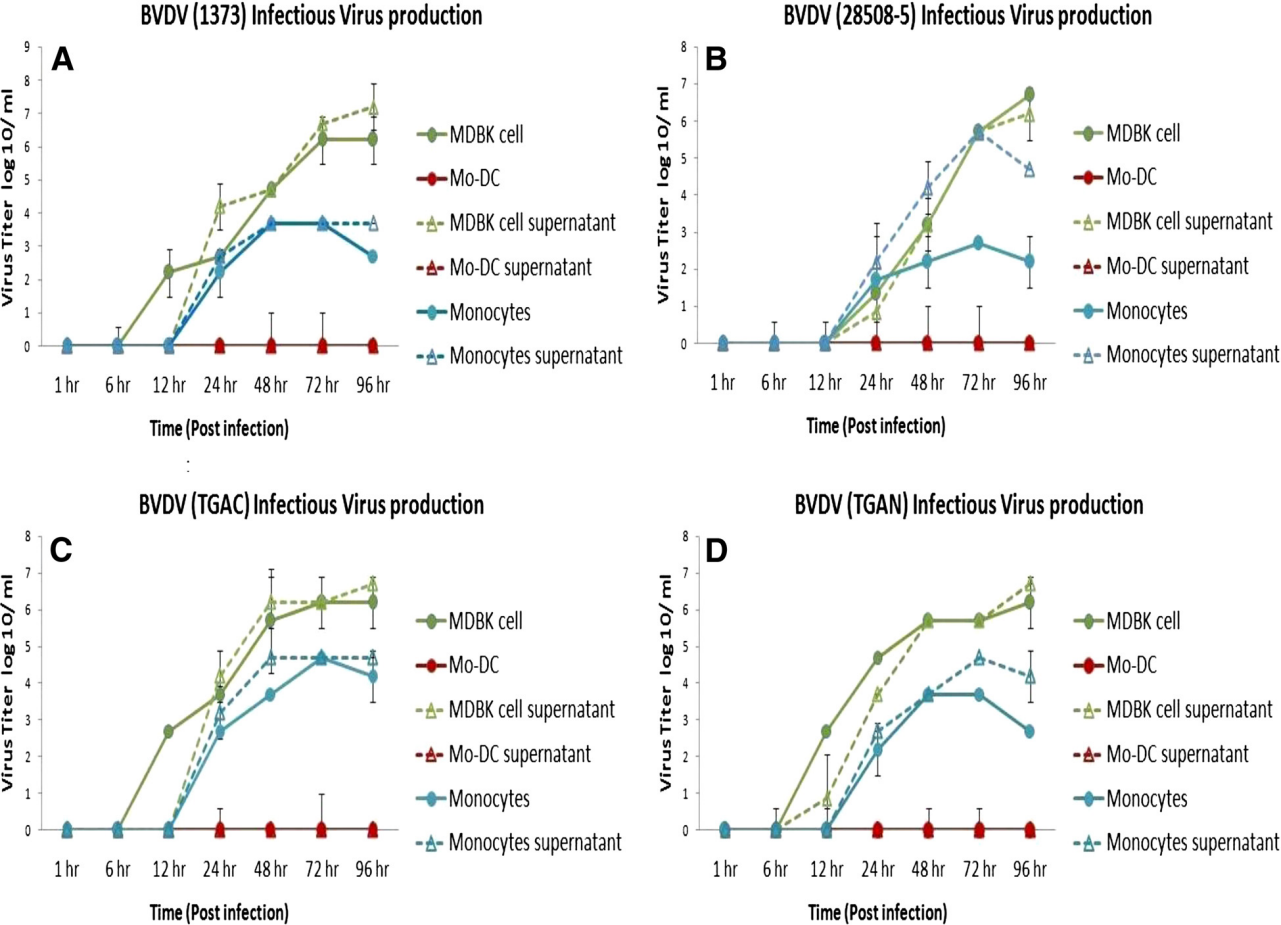
**Virus production by Mo-DC, monocytes, and MDBK cells.** The Mo-DC, monocytes, or MDBK cells were infected with ncp BVDV2a 1373 **(A)**, ncp BVDV2a 28508-5 **(B)**, cp BVDV1b TGAC **(C)**, or ncp BVDV1b TGAN **(D)** at an MOI of 6. Cells and supernatants were collected at 1, 6, 12, 24, 48, 72, and 96 hour(s) post-infection and analyzed for viral titer. Each experiment was repeated three times and mean virus titer was calculated at each time point.

**Figure 6:**
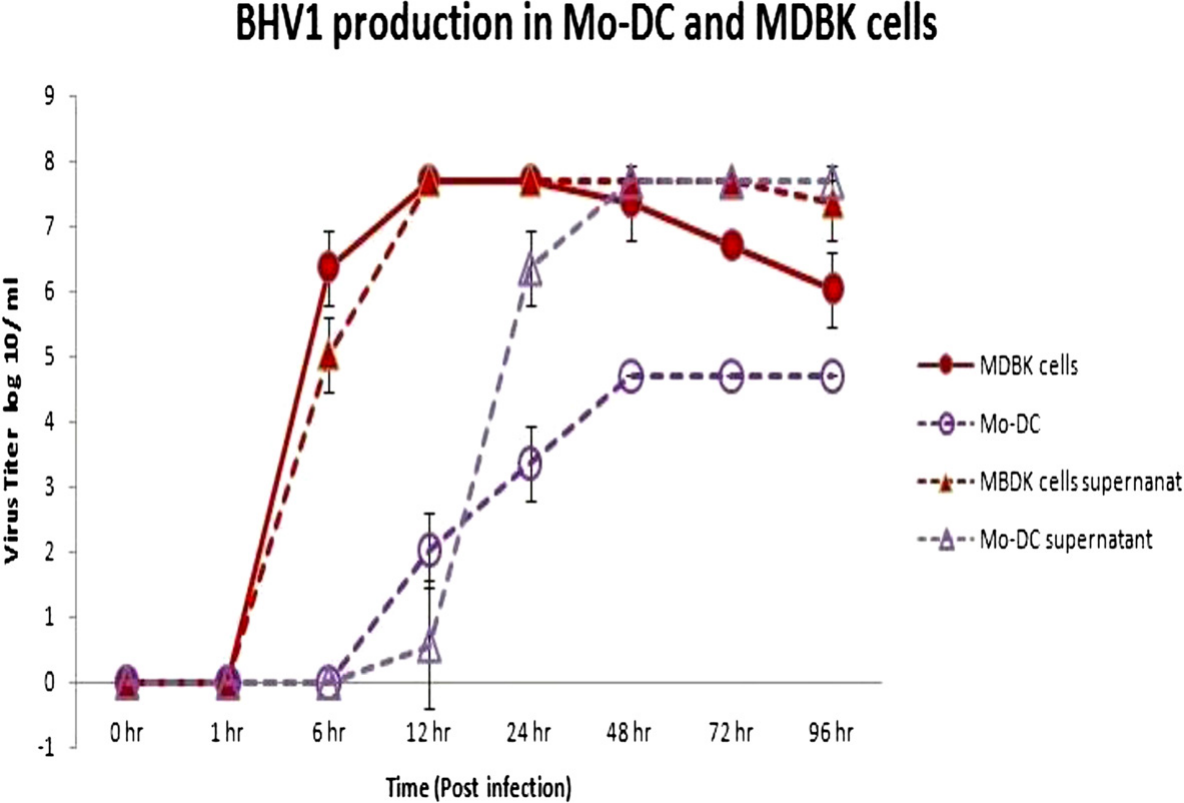
**BHV1 production by Mo-DC.** The Mo-DC or MDBK cells were infected with the Cooper strain of BHV1 at an MOI of 6. Cells and supernatants were collected at 1, 6, 12, 24, 48, 72, and 96 hour(s) post-infection and analyzed for viral titer. The experiment was repeated three times and mean virus titer was calculated for both cells at each time point.

Following 7 days of culture to differentiate monocytes to Mo-DC (fully differentiated Mo-DC) freshly collected monocytes, and Mardin-Darby Bovine Kidney (MDBK; positive control) cells were infected with one of the four BVDV strains (i.e., ncp BVDV2a 1373, ncp BVDV2a 28508-5, ncp BVDV1b TGAN, or cp BVDV1b TGAC strain at an MOI of 6). Production of BVDV was measured at 1, 6, 12, 24, 48, 72, and 96 hour(s) post-infection. The virus titer in supernatant or cell lysates was measured as described by Reed and Muench [9]. No infectious BVDV was produced by Mo-DC, regardless of strain used, while monocytes and MDBK cells supported production of infectious BVDV. Specifically, the BVDV titers in MDBK cells for ncp BVDV2a 1373 peaked at 6.2 ± 0.71 log10/ml at 72 hours post-infection (Figure 5A); ncp BVDV2a 28508-5 titers peaked at 6.7 ± 0.0 log10/ml at 96 hours post-infection (Figure 5B); cp BVDV1b TGAC titers peaked at 6.2 ± 0.71 log10/ml at 72 hours post infection (Figure 5C); and ncp BVDV1b TGAN titers peaked at 6.2 ± 0.71 log10/ml at 96 hours post infection (Figure 5D). Furthermore, BVDV titers in freshly collected monocytes peaked at 3.67 ± 0.71 log10/ml for ncp BVDV2a 1373 at 48 hours post infection (Figure 5A); ncp BVDV2a 28508-5 titers peaked at 2.7 ± 0.0 log10/ml at 72 hours post-infection (Figure 5B); cp BVDV1b TGAC titers peaked at 4.7 ± 0.00 log10/ml at 72 hours post infection (Figure 5C); and ncp BVDV1b TGAN titers peaked at 3.7 ± 0.001 log10/ml at 48 hours post-infection (Figure 5D). The BVDV infected Mo-DC did not produce infectious virus (i.e., titers of 0.0); however, Mo-DC supported production of the Cooper strain of bovine herpesvirus 1 (BHV-1), indicating that the failure of Mo-DC to produce virus was BVDV specific. The BHV-1 titer peak was 4.7 ± 0.0 log10/ml at 48 hours post-infection (Figure 6).

### Mo-DC lose the ability to produce infectious BVDV during differentiation

**Figure 7:**
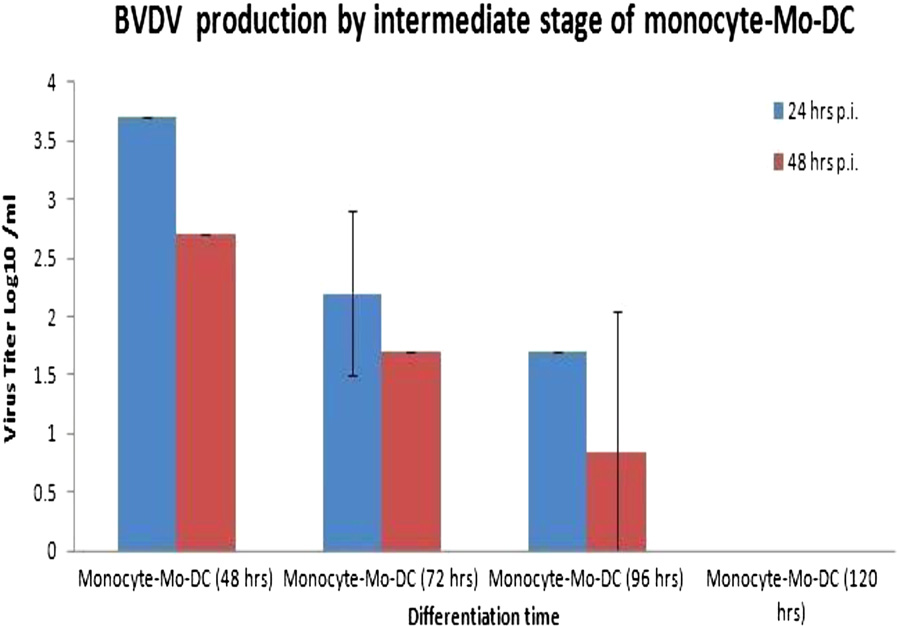
**Virus production of ncp BVDV2a-1373 strain of BVDV by intermediate stages of cells differentiating from monocyte to Mo-DC.** Cells in the intermediate stages of differentiation were harvested at 48, 72, 96, and 120 hours. Cells at each intermediate stage of differentiation were infected with ncp BVDV2a 1373 strain of BVDV with a 6 MOI. The cells were collected at 24 and 48 hours post-infection and analyzed for viral titer. The experiment was repeated three times and mean virus titer at each time point.

Cells were collected during the intermediate stages of differentiation from monocytes to Mo-DC (i.e., at 48, 72, 96, and 120 hours) and infected with ncp BVDV2a 1373 at an MOI of 6. The cells were then collected at 24 or 48 hours post-infection and analyzed for virus titer in cell lysate, as described by Reed and Muench [9]. Results indicated that the ability of Mo-DC to produce virus is lost during differentiation, and production of infectious virus was completely halted by 120 hours of differentiation. At 48 hours of differentiation from monocyte to Mo-DC, cells produced 3.69 ± 0.00 log10/ml and 2.69 ± 0.00 log10/ml infectious BVDV at 24 and 48 hours post-infection, respectively; whereas, at 72 hours of differentiation, the intermediate monocyte to Mo-DC produced 2.19 ± 0.71 log10/ml and 1.68 ± 0.00 log10/ml BVDV at 24 and 48 hours post-infection, respectively. At 96 hours of differentiation, BVDV titers from Mo-DC were comparatively less (i.e., 1.69 ± 0.00 log10/ml and 0.85 ± 1.20 log10/ml at 24 and 48 hours post-infection, respectively), and no virus was produced by Mo-DC at 120 hours of differentiation at 24 and 48 hours post-infection (Figure 7).

### BVDV replicates viral RNA in Mo-DC

**Figure 8:**
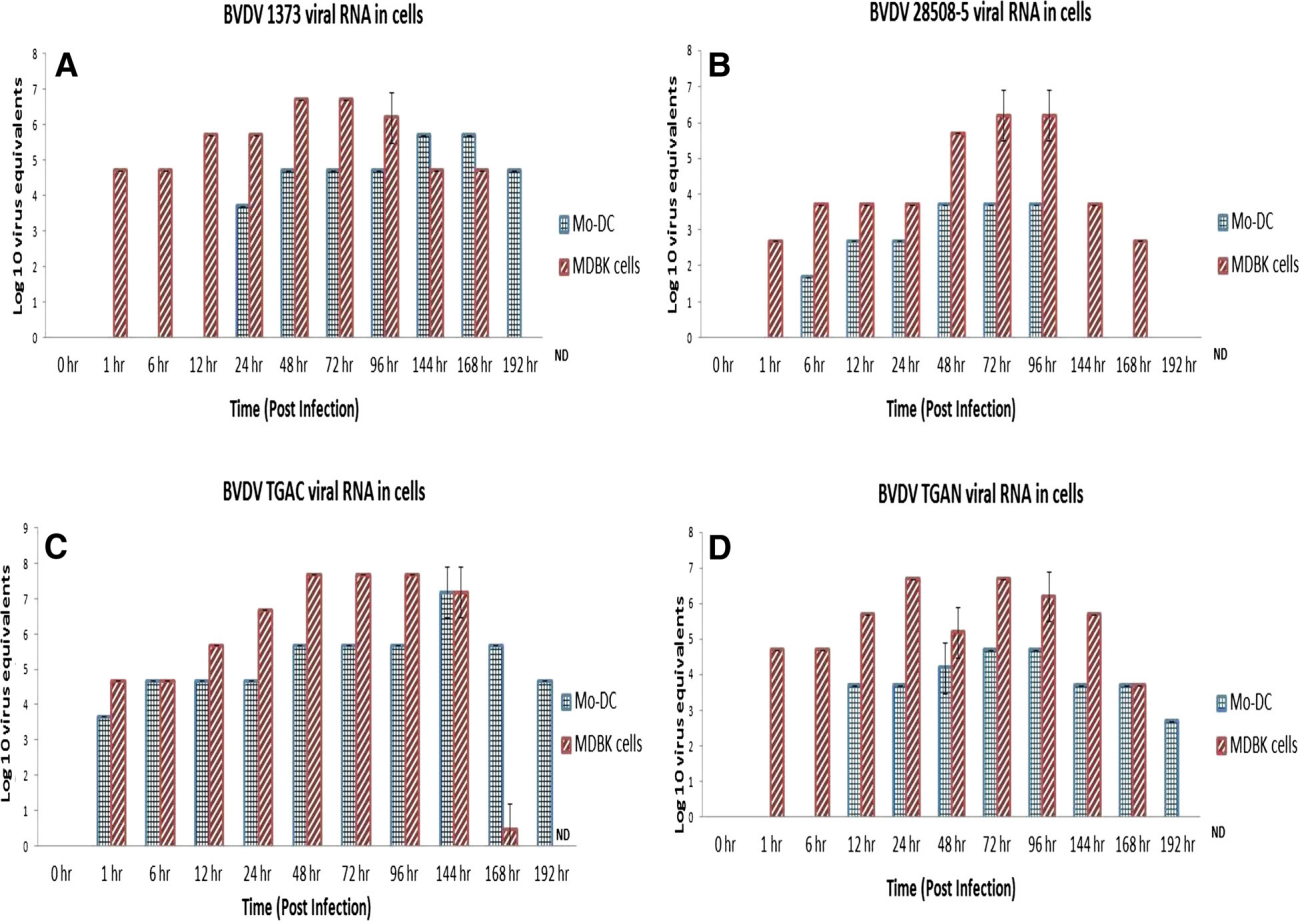
**Replication of BVDV viral RNA in Mo-DC or MDBK cells.** Mo-DC or MDBK cells were infected with **A)** ncp BVDV2a 1373, **B)** ncp BVDV2a 28508-5, **C)** cp BVDV1b-TGAC, or **D)** ncp BVDV1b TGAN strains of BVDV at an MOI of 6. Cells were collected at 0, 1, 6, 12, 24, 48, 72, 96, 144, 168 and 192 hour(s) post-infection. Viral RNA was extracted from the cells at each time point and quantified using qRT-PCR at each time point. The experiments were repeated three times and mean value was calculated at each time point.

Mo-DC were not able to produce infectious BVDV; therefore, the ability of Mo-DC to produce BVDV RNA was examined. Viral RNA was extracted from BVDV-infected Mo-DC at 0, 1, 6, 12, 24, 48, 72, 96, 144, 168, and 192 hours post-infection. Viral RNA was quantified at each time point using quantitative real-time polymerase chain reaction (qRT-PCR) [10]. Testing indicated that viral RNA replicated in Mo-DC. Interestingly, the kinetics of viral RNA production differed between viral strains. Specifically, replication of viral RNA for the ncp BVDV2a 1373 strain began, peaked, and started to decline at 24, 144, and 192 hours post-infection, respectively (Figure 8A); replication of cp BVDV2a 28508-5 viral RNA began at 6 hours, began to decline at 96 hours, and was no longer producing viral RNA at 144 hours post-infection (Figure 8B); replication of the cp BVDV1b TGAC viral RNA began, peaked, and started to decline at 1, 144, and 168 hour(s) post-infection, respectively (Figure 8C); and production of the ncp BVDV1b TGAN viral RNA began replicating at 12 hours, peaked at 72 hours, and began to decline at 144 hours post-infection.

**Figure 9:**
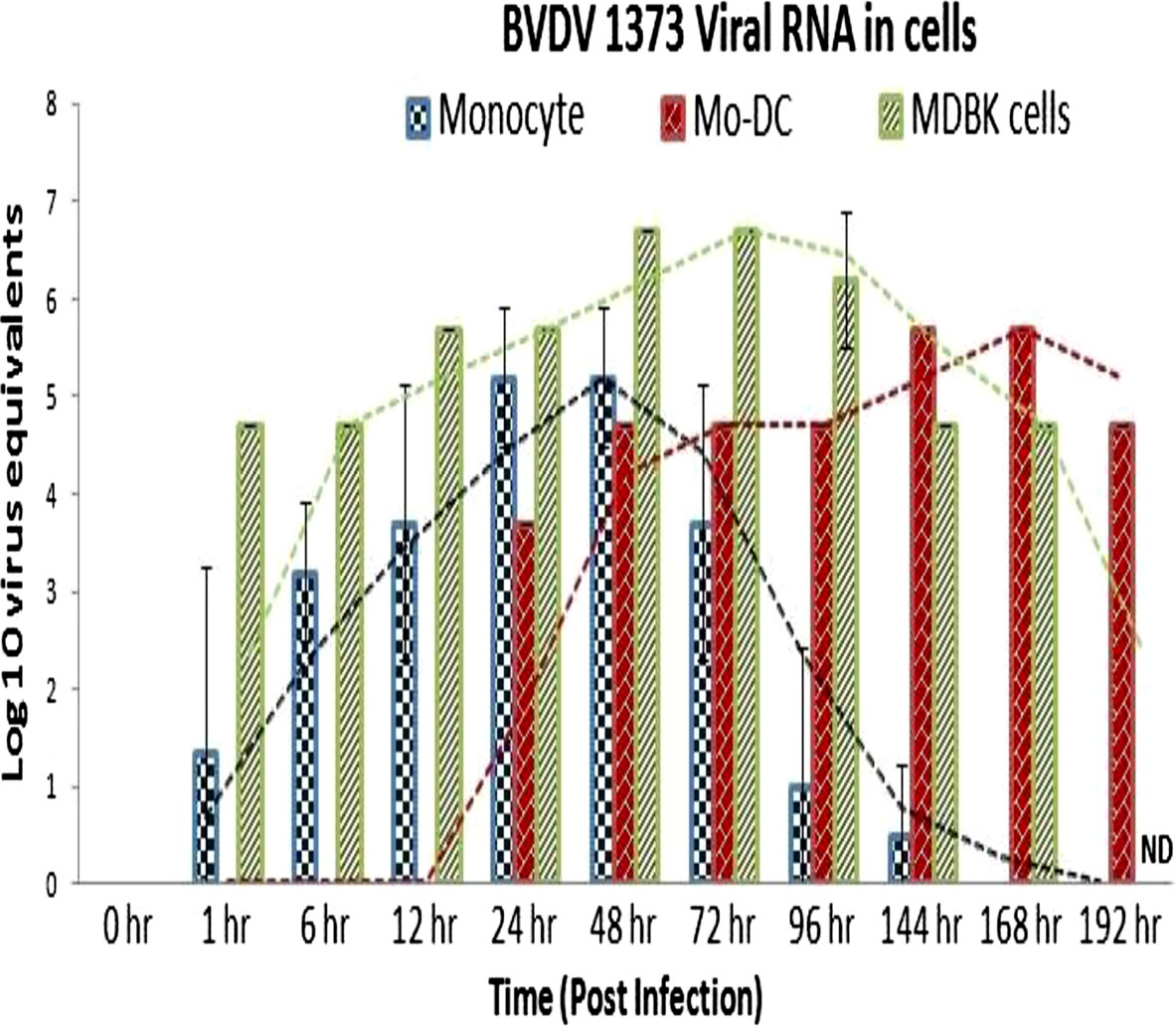
**Replication of ncp BVDV2a-1373 viral RNA in Monocytes, Mo-DC, or MDBK cells.** Monocytes, MDBK cells, or Mo-DC were infected with ncp BVDV2a-1373 at 6 MOI. Cells were collected at 0, 1, 6, 12, 24, 48, 72, 96, 144, 168 and 192 hour(s) post-infection. Viral RNA was extracted from cells at each time point and quantified using qRT-PCR. The experiments were repeated three times and mean value was calculated at each time point.

Comparisons were made between replication of BVDV RNA in monocytes and Mo-DC. Freshly collected monocytes were infected with ncp BVDV2a 1373 at an MOI of 6. The viral RNA was extracted from infected monocytes at 0, 1, 6, 12, 24, 48, 72, 96, 144, 168, and 192 hour(s) post-infection and was quantified at each time point using qRT-PCR. Results showed that BVDV viral RNA started to replicate in monocytes as early as 1 hour post-infection and had ceased at 168 hours post-infection; whereas replication of viral RNA in Mo-DC began at a later time point and had a longer duration (i.e., 24 through 168 hours post-infection) (Figure 9).

### Mo-DC produced the BVDV non-structural (NS5A) and envelope (E2) viral proteins

**Figure 10:**
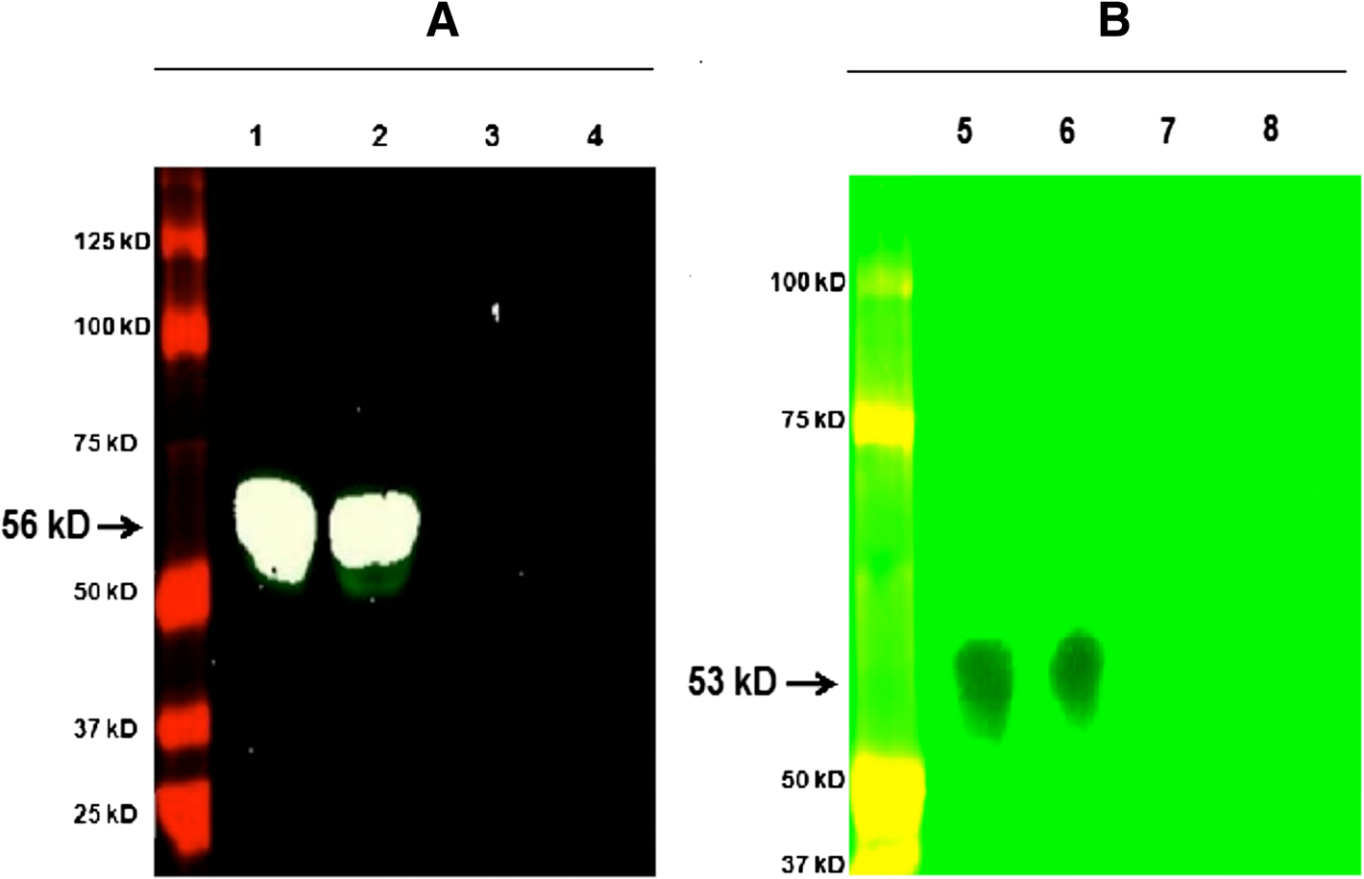
**Presence of NS5Aor E2 viral protein of BVDV in BVDV Infected Mo-DC.** Mo-DC (lane 1 and 6) or MDBK cells (lane 2 and 5) were infected with ncpBVDV1a-1373 with MOI of 6. The mock-infected Mo-DC (lane 3 and 8) or MDBK cells (lane 4 and 7) were used as negative control. Cells were collected 72 hours post-infection. The Western Blot was performed using harvested cell lysate for BVDV NS5A or E2 viral proteins. Specific NS5A protein **(panel A)** with band size of 56 kD or E2 protein **(panel B)** with band size of 53 kD in MoDC or MDBK cell lysate, infected with ncp BVDV2a-1373 were observed.

Viral RNA replicated in Mo-DC, but infectious virus was not produced; therefore, to determine whether or not the RNA was translated into viral proteins, a Western Blot was performed. The test was completed using lysate harvested at 72 hours from Mo-DC or MDBK cells infected with ncp BVDV2a 1373 at an MOI of 6. The ncp BVDV2a 1373 infected MDBK cells were used as positive control while mock infected Mo-DC or MDBK cells were used as negative control. The results showed that Mo-DC lysate contained NS5A (Figure 10A) or E2 (Figure 10B), indicating that the infected Mo-DC produced viral proteins but did not release infectious virus.

### The cp biotype up-regulated and ncp biotypes down-regulated MHCI, MHCII, and CD86 expression on Mo-DC

Following infection or mock-infection (i.e., control cells) with the four strains of BVDV, the Mo-DC were stained for MHCI, MHCII, or CD86 and fixed at 0, 1, 6, 12, 24, 48, and 72 hour(s) to determine the effects of infection on cell surface expression. The mean fluorescent intensity (MFI) in mock-infected Mo-DC at 0 hours was utilized as 100%, the mock-infected Mo-DC at each time point were used as control, and the percent change at each time was calculated.

**Figure 11:**
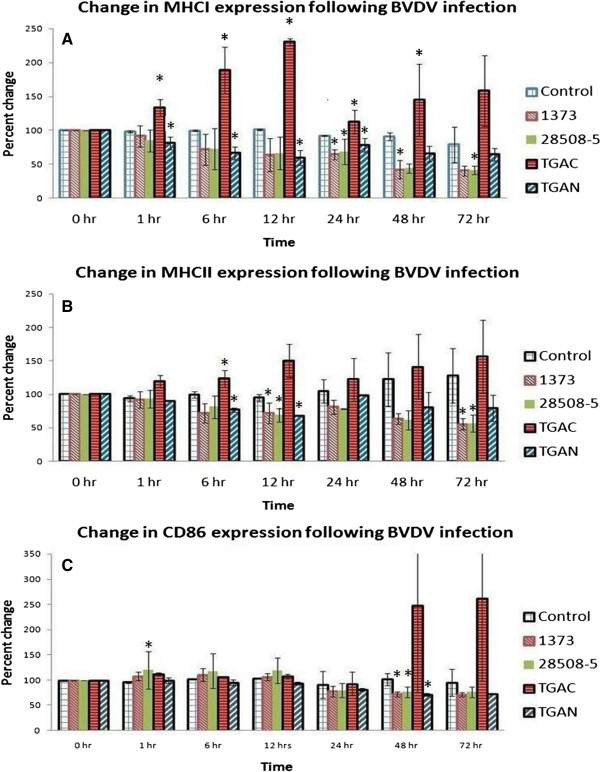
**Effect of BVDV on Mo-DC cell surface marker expression.** Differentiated Mo-DC were infected with either of four strains of BVDV, including ncp BVDV2a 1373, ncp BVDV2a 28508-5, or a homologous pair of ncp or cp BVDV1b viruses (ncp BVDV1b TGAN or cp BVDV1b TGAC) with an 6 MOI. The cells were stained for **A)** anti-MHCI; **B)** anti-MHCII or **C)** anti-CD86 antibody at 1, 6, 12, 24, 48, and 72 hour(s) post-infection. The mock-infected Mo-DC) at each time point was used as control (white legend). The mean fluorescent intensity (MFI) of expression was analyzed using flow cytometry. The each experiment was repeated three times. Asterisks (*) indicate significant difference from controls (*P* < 0.05).

It was determined that MHCI expression in Mo-DC infected with cp BVDV1b TGAC increased with the course of infection. The expression of MHCI was up-regulated from 100% at 0 hour to 133.50 ± 11.88% (i.e., approximately 33%), 189.10 ± 34.3% (i.e., roughly 89%), 230.2 ± 5.22%, (i.e., about 130%), 112.22 ± 17.29% (i.e., approximately 12%), 144.60 ± 53.14% (i.e., about 44%), and 158.16 ± 52.43% (i.e., roughly 58%) at 1, 6, 12, 24, 48, and 72 hours post-infection, respectively, as compared to time point controls (i.e., mock-infected Mo-DC). The MHCI up regulation at 1, 6, 12, 24 or 48 hours post-infection was significantly different then mock-infected control (P < 0.05) (Figure 11A). In contrast, MHCI expression was down-regulated following infection with ncp BVDV strains as compared to mock-infected controls. The expression of MHCI was down-regulated from 100% at 0 hour to 64.15 ± 9.25% (i.e., about 36%), 41.30 ± 5.95% (i.e., about 59%) and 40.10 ± 7.18% (i.e., approximately 60%) at 72 hours post-infection following ncp BVDV1b TGAN, ncp BVDV2a 28508-5 or ncp BVDV2a 1373 infection respectively (Figure 11A). The ncp BVDV1b TGAN infection to Mo-DC significantly down-regulated the MHCI expression on Mo-DC at 1, 6, and 12 and 24 hour(s) post-infection as compared to mock-infected control (P > 0.05). The ncp BVDV1a 28508-5 significantly (P < 0.05) down-regulated the MHCI expression on Mo-DC at 24 and 72 hr post infection while the ncp BVDV1a 1373 infection down-regulated MHCI expression at 24 and 48 hours post-infection (P < 0.05) (Figure 11A).

Expression of MHCII increased during the course of infection in Mo-DC infected with cp BVDV1b TGAC. MHCII expression increased in cp BVDV1b TGAC infected Mo-DC from 100% at 0 hour to 156.83 ± 54.48% (i.e., roughly 56%) at 72 hours post-infection The MHCII expression on Mo-DC following 6 hours infection with cp BVDV1b TGAC was significantly higher than its time point mock-infected control (P > 0.05) (Figure11B).

In contrast, MHCII expression was down-regulated in Mo-DC, infected with ncp BVDV strains. The ncp BVDV1b TGAN infection reduced the MHCII expression from 100% at 0 hour to 79.33 ± 19.33% (around 21%) at 72 hours post-infection. Similarly, expression of MHCII was reduced from 100% at 0 hour to 56.44 ± 13.01% (i.e., about 44%) and 55.10 ± 8.39% (i.e., approximately 45%) at 72 hours post infection following ncp BVDV2a 28508-5 or ncp BVDV2a 1373 infection respectively. The MHCII expression on Mo-DC was significantly down-regulated at 6 and 12 hours post-infection with ncp BVDV1b TGAN while ncp BVDV2a 28508-5 or ncp BVDV2a 1373 infection significantly down-regulated the MHCII expression on Mo-DC at 12 and 72 hours post-infection as compare to their time point control Mo-DC (P > 0.05) (Figure 11B).

Patterns of expression of CD86 in Mo-DC were similar to those of MHCI and MHCII in regards to the BVDV biotypes examined, as the cp biotype up-regulated and the ncp biotypes down-regulated CD86 expression. Expression of CD86 in cp BVDV1b TGAC infected Mo-DC was up-regulated from mock-infected controls as 100% at 0 hour to 261.22 ± 162.00 (i.e.160%) at 72 hours post-infection (Figure 11C). Conversely, expression of CD86 was decreased in Mo-DC infected with ncp BVDV1b TGAN, ncp BVDV2a 28508-5 or ncp BVDV2a 1373 from 100% at 0 hour to 72.64 ± 1.38% (i.e. 28%), 76.31 ± 11.03% (i.e., about 24%) or 71.36 ± 4.08% (i.e., roughly 29%) at 72 hours post-infection, respectively. The down-regulation of CD86 in ncp BVDV1b-TGAN, ncp BVDV2a 28508-5 or ncp BVDV2a 1373 infected Mo-DC was significant at 48 hours post-infection as compare to their time point control (Figure 11C).

## Discussion

This study revealed that Mo-DC and its progenitor cells (i.e., monocytes) could become infected with the BVDV virus; and while monocytes subsequently produced infectious virus, Mo-DC did not. Furthermore, virus production decreased with monocyte differentiation to Mo-DC and had completely ceased by120 hours of differentiation; thus, monocytes lost the ability to produce infectious virus with differentiation to Mo-DC. It was also determined that Mo-DC supported BVDV viral RNA replication but; the kinetics of viral RNA production was different between different viral strains. Additionally, viral proteins were translated in BVDV infected Mo-DC; thus, the accumulation of BVDV viral RNA and its subsequent translation into viral proteins suggest that failure to produce infectious virus may be the result of a hindrance in viral assembly, packaging, or release in Mo-DC. Further studies using electron microscopy may confirm the actual level of hindrance in infectious virus production.

A previous study that utilized a homologous pair of BVDV ncp and cp viruses (i.e., Pe515ncp and Pe515cp) revealed that Mo-DC were able to replicate and produce infectious BVDV [11]. The difference in the ability of the Mo-DC to produce infectious virus between that study and the present study may be due to the use of different monocyte isolation and Mo-DC culturing methods, as the previous study utilized CD14^+^Mo-DC, while the current study examined Mo-DC that were determined to be non-adherent CD14^-^ cells. CD14 is a cell surface marker specifically for monocytes and macrophages. It acts as a co-receptor for bacterial lipopolysaccharide (LPS) with toll-like receptor- 4 (TLR 4) [12]. CD14 expression is reduced during differentiation of monocytes to Mo-DC [13,14]. Likewise, in another study, bovine CD11b^+^Mo-DC also produced infectious BVDV; however, that study did not describe the culturing method, phenotype, or morphology of the Mo-DC used [15]. CD11b is an adhesion molecule and complement receptor that is expressed on neutrophils, monocytes, macrophages and Mo-DCs [16,17]. The above cell surface molecules in combination with DEC205 are routinely used to characterize the Mo-DC. DEC205 is a specific marker of Mo-DC [18].

A number of studies have been performed using Mo-DC and hepatitis C virus (HCV; another member of the *Flaviviridae* family). Similarly, both BVDV and HCV have a similar structural organization, can cause chronic infections in their respective hosts, utilize the low-density lipoprotein (LDL) receptor to enter the host cells, and use a functionally similar internal ribosome entry site (IRES) for translation. In addition, both viruses use NS5B RNA-dependent RNA polymerase and have a similar mechanism for virion maturation, assembly, and release [19]. In one study, the J6/JFH strain (a chimeric HCV of genotype 2a) did not replicate in B or T lymphocytes, monocytes, macrophages, or dendritic cells, while it did replicate and produce infectious virus in Huh-7.5 cells [20]; whereas, another study found that Mo-DC infected with the JFH1 strain of HCV with an MOI of 1did not support HCV RNA replication or antigen production [21]. In contrast to the present study, the mechanism responsible for the inability to produce infectious HCV was different, as BVDV viral RNA was replicated and translated into viral protein. This variation in HCV and BVDV replication may be due to their replication requirements, as HCV tropism is restricted to hepatocytes of humans and chimpanzees [22], while BVDV can replicate in epithelial and non-epithelial cells [23].

A trial utilizing influenza virus in mouse bone marrow-derived DC resulted in abortive replication. However, viral genome transcription and replication occurred for each gene segment along with the translation of viral hemagglutinin and nucleoprotein. Further the study using electron microscopy examination revealed that the resulting failure was associated with defective viral release, possibly due to insufficient synthesis or stability of one or more viral proteins required for release of the virus [24]. Similarly, the current study showed the abortive replication of BVDV in Mo-DC. The exact mechanism of abortive BVDV replication in Mo-DC is not known. However future ultrastructural examination of BVDV infected Mo-DC using electron microscopy could be intriguing to understand the stage at which production of infectious virus is blocked (i.e. assembly, egress etc).

The current study showed that Mo-DC precursor monocytes produced infectious BVDV while Mo-DC failed to do so. It would be interesting to know the dynamics of Mo-DC differentiation/maturation and viral production. Previous studies have demonstrated that Mo-DC differentiated with GMCSF and IL-4 were immature and could be matured by proinflammatory cytokines, CD40L, IFN-γ or LPS [13,25]. There has been little work done on effect of BVDV strains on Mo-DC maturation. While it is well established that cp BVDV induced more IFN-γ than ncp BVDV in vitro [26,27], it is likely that cp BVDV strains induce Mo-DC maturation more than ncp BVDV strains in vitro. To confirm this hypothesis, a future study would be helpful using different BVDV strains along with lipopolysaccharide (LPS) as maturation differentiation control.

One of the factors that may affect the production of virus in Mo-DC is interaction with other cell populations. In this *in vitro* study, isolated cell populations were used; whereas, testing in the *in vivo* environment may result in different outcomes. Interaction of Mo-DC with T cells *in vivo* may trigger the production of infectious virus. In one study, the interaction of antigen presenting cells (APC) with T cells enhanced HCV infection, as the interaction of APC with T cells led to activation of T cells [27]. Activated T cells produce interleukin-2 (IL-2), a substance that enhances CD81 expression in thymocytes [28]. Expression of CD81 on thymocytes facilitates the entry of HCV into cells, and thus, its replication [29]. Similarly, co-stimulation through interaction of CD28 and B7 increased HIV type 1 replication [30]. Therefore, it stands to reason that other cell populations could be affected. For example, the macrophage may become infected by phagocytizing the apoptotic Mo-DC containing viral RNA and may then begin producing infectious BVDV. As these probabilities are theoretical, exploration using T cell activation assay or *in vivo* trials would be beneficial.

The cell susceptibility can greatly be influenced with changes in its surface receptor expression, particularly receptors that mediate virus entry. BVDV infects cells via Receptor-mediated endocytosis using the LDL receptor [19]. Given that oxidized LDL has been demonstrated to promote differentiation of dendritic cells from monocytes in other species [31], it would be interesting to explore the dynamics of LDL receptor expression during monocyte to Mo-DC differentiation with respect to cell infectivity and virus production. These future studies could also explore the effect of different BVDV on LDL expression for better understanding of the BVDV pathogenesis.

In the current study, infection with BVDV affected cell surface marker expression on Mo-DC. The cp BVDV1b-TGAC strain enhanced MHCI, MHCII, and CD86 expression, while ncp strains of BVDV (i.e., ncp BVDV2a 1373, ncp BVDV2a 28508-5, and ncp BVDV1b TGAN) reduced the MHCI, MHCII, and CD86 expression during the course of infection. In another study that used bovine monocyte-derived macrophages (MDM), MHCI was up-regulated following infection with cp BVDV and was down-regulated in cells infected with a ncp strain of BVDV, while both cp and ncp strains down-regulated the MHCII expression in MDM [32]. The difference in MHCII results between that study and the present study may be due to a difference in the cell types chosen for analysis, as the Mo-DC reported here were non-adherent CD14^-^ cells, while the MDM were adherent CD14^+^ cells. A study using differential detergent fractionation (DDF) analysis of bovine monocytes showed that 53 bovine proteins involved in the immune function of professional APC were altered following BVDV infection. These altered molecules include adhesion 151 molecules, toll-like receptors (TLR 1, 6, and 8), antigen uptake, MHC I and II, cytokines, and growth factor synthesis molecules [33]. The above study supported the current finding that showed the alteration of MHCI, MHCII and CD86 expression in BVDV infected Mo-DC. The above study also showed the alteration of cell adhesion molecules and capability of antigen uptake by APC, that may affect the Mo-DC infectivity and ultimately virus producing capability that need to be explored.

The changes noted in surface marker expression on Mo-DC could be due to differences in interferon expression between cp and ncp biotypes of BVDV. Type 1 IFN plays an important role in up-regulation of MHCI expression [34], thus, reduced type 1 IFN production in Mo-DC infected with ncp BVDV may explain the down-regulation of MHCI and MHCII. Similarly, up-regulation of MHCI and MHCII in Mo-DC infected with cp BVDV may be due to an increase in type 1 IFN [26,35]. It would be interesting to validate the effect of Type 1 IFN on cell surface marker expression by using poly I:C treated cells as control. The previous study showed that ncp BVDV inhibited of IFN induction was a result of blocking the IRF-3 pathway [27]. It may be possible that BVDV affects other pathways such as the melanoma differentiation-associated gene 5 (MDA5) signaling pathway, which will need to be explored for better understanding of BVDV pathogenesis. Alternatively, infection may have a direct effect on MHC folding and assembly, and consequently, expression. For example, infection with HCV changed MHCI expression in infected cultured cancerous cells lines (i.e., human hepatoma and mouse lymphoma cells) via disruption in MHCI protein folding and assembly [36-38]. This study found that HCV replicons induced endoplasmic reticulum (ER) stress that resulted from a decline in protein glycosylation. The decrease in protein glycosylation disrupted protein folding and prevented the assembly of MHCI molecules. Reduced assembly of MHCI molecules ultimately resulted in MHCI down-regulation. Similarly, Human Immunodeficiency Virus (HIV) was shown to reduce MHCI and CD86 expression via the Nef protein in a human embryonic kidney cell line (293 T), the human monocytic U937 cell line, as well as in mouse macrophages and dendritic cells. The Nef protein binds and endocytoses MHCI molecules by the ARF6 pathway, thus resulting in reduced MHCI expression [39,40]. The mechanism by which BVDV infection alters cell surface marker expression needs to be explored, as this information would provide a better understanding of immunosuppression caused by BVDV.

## Conclusions

Findings from the current study demonstrated that Mo-DC failed to produce infectious BVDV. The ability to produce infectious BVDV was reduced with differentiation of monocyte to Mo-DC and completely stopped at 120 hours of differentiation. However, BVDV viral RNA replicated and was translated into viral proteins in Mo-DC, indicating that inability to produce infectious virus may be due to assembly or release of virus. Infection of Mo-DC with the cp strain of BVDV up-regulated expression, while infection with ncp strains of BVDV down-regulated expression of the cell surface markers MHCI, MHCII, and CD86. The up-regulation of cell surface marker expression that results from cp BVDV infection may explain why a better immune response is induced when modified live virus vaccines that contain cp strains of BVDV are used. On the other hand, the down-regulation of cell surface marker expression by ncp strains of BVDV may be an important function of immunosuppression and subsequent development of persistent infection.

## Methods

### Animals

Brown Swiss calves (8-12 months of age) were used in this study. All animals were deemed healthy prior to use and were housed at the Dairy Farm at South Dakota State University, Brookings, SD, U.S.A. Animal handling and blood collection were approved (approvals # 09-039E and 12-052A) and performed according to the guidelines of the SDSU Institutional Animal Care and Use Committee.

### Isolation of monocyte and differentiation into Mo-DC (Monocyte-derived dendritic cell)

Peripheral blood mononuclear cells (PBMC) were isolated from the whole blood of healthy calves using gradient centrifugation using 65% percoll (GE Healthcare Biosciences, Pittsburgh, PA, USA. PBMC were cultured at 37°C in 6-well plates using Roswell Park Memorial Institute medium (RPMI) 1640 medium supplemented with 10% fetal bovine serum (FBS), penicillin (100 U/ml) and streptomycin (100 μg/ml) for three hours. The adherent cells were harvested using Accutase (eBioscience, San Diego, CA, USA) and confirmed as MHCI^+^, MHCII^+^, and CD14^+^ monocytes. The monocytes were cultured in RPMI 1640 medium supplemented with 20% FBS, 1 mM sodium pyruvate, penicillin (100 U/ml), streptomycin (100 μg/ml), recombinant bovine GM-CSF (100 ng/ml) and IL-4 (200 ng/ml) for 7 days. A 750 μl medium supplemented with cytokine was added to each well on alternate days, and the loosely attached or floating Mo-DC were harvested on day 7 and were confirmed morphologically and phenotypically. Morphologically, Mo-DC were 4 to 5 times larger than monocytes and had long dendrites. Phenotypically, Mo-DC were determined as MHCI^+^, MHCII^+^, CD86^+^, CD205^+^, CD14^-^, and CD21^-^.

### Virus

The Cooper strain of bovine herpesvirus1 (BHV1) and four strains of BVDV [i.e., a homologous pair of ncp and cp viruses (ncp BVDV1b TGAN and cpBVDV1b TGAC) recovered from an animal that died of mucosal disease; the severe acute ncp BVDV2a 1373); and the moderate acute ncp BVDV2a 28508-5 strain] were used in this study. All the BVDV virus stock used in the study was kindly provided by Dr. Julia F Ridpath (Ruminant Diseases and Immunology Research Unit, National Animal Disease Center, USDA, Ames, IA, USA).

### Cells

Passage 95–110 of BVDV free MDBK cells, grown in minimal essential medium (MEM, Gibco BRL, Grand Island, NY) at a pH of 7-7.4, and supplemented with 10% BVDV free fetal calf serum (PPA, Pasching, Austria), penicillin (100 U/ml), and streptomycin (100 μg/ml) were used in the study.

### Virus titration

Freshly collected monocytes, fully differentiated Mo-DC, and MDBK cells were infected with ncp BVDV2a 1373, ncp BVDV2a 28508-5, cp BVDV1b TGAC, or ncp BVDV1b TGAN at an MOI of 6. The cell lysate or supernatant were collected at 0, 1, 6, 12, 24, 48, 72, and 96 hour(s) post-infection. Virus titers in cell lysate or supernatant at each time point were determined as described by Reed and Muench [9]. Additionally, cells were infected with BHV1 at the same MOI and were used as positive control for virus replication at the same time points.

To determine at which time point of differentiation Mo-DC lose infectious virus production capacity, the monocytes, cells of intermediate stages at 48, 72, 96 and 120 hours of differentiation were infected with ncp BVDV2a 1373 at an MOI of 6. The cells were collected at 24 and 48 hours post-infection and analyzed for virus titer in cell lysate.

### Viral RNA isolation and quantification

Fully differentiated Mo-DC and MDBK cells were infected with ncp BVDV2a 1373, ncp BVDV2a 28508-5, cp BVDV1b TGAC, or ncp BVDV1b TGAN at an MOI of 6. Cells were collected at 1, 6, 12, 24, 72, 96, 120, 144, 168, and 192 hour(s) post-infection. Viral RNA was isolated from infected cells using the QIAamp Viral RNA Mini kit (Qiagen, Valencia, CA, USA) and was quantified by qRT-PCR (Stratagene MX3000P Real-Time Thermocycler, Stratagene Inc., La Jolla, USA) in 25 μl reaction with 6-carboxy-fluorescein (FAM) dye. The qRT-PCR targeted the 5′ untranslated region (5′UTR) of viral genome using the following primers: forward = 5-GGGNAGTCGTCARTGGTTCG-3; and reverse = 5-TGCCATGTACAGCAGAGWTTTT-3) [10].

To compare the viral replication dynamics in monocytes, fully differentiated Mo-DC and MDBK cells, the cells were infected with ncp BVDV2a1373. Viral RNA was isolated and quantified using qRT-PCR, as described above. The RNA extracted from non-infected cells was used as internal control at each reaction.

### Western blot

To determine whether BVDV viral RNA was translated into viral proteins in Mo-DC, a Western blot was performed targeting one structural protein (envelope protein -2: E2) that play a major role in virus attachment and entry to host cell [41] and non-structural protein 5A (NS5A), that interacts with the host cellular protein and inhibits NF-B activation [42]. Western blot was performed as described by Devireddy and Jones [43], with some modifications. The MDBK cells or Mo-DC were infected with ncp BVDV2a 1373 at an MOI of 6for 72 hours, washed with PBS, and then lysed with 200 μl of RIPA buffer containing the complete ULTRA tablet containing protease inhibitor (Roch Diagnostic, GmbH, Sandhofen, Germany). The mock infected MDBK cells or Mo-DC were used as control. The cell lysate was centrifuged at 13,000 rpm for 5 minutes and the supernatant was collected. Forty μl of cell lysate was loaded in 12.5% sodium dodecyl sulfate -polyacrylamide gel electrophoresis (SDS-PAGE) and the dual color protein ladder (10-250kD; Bio-Rad, Hercules, CA, USA) was used as a standard. The proteins were transferred onto a nitrocellulose membrane (Whatman GmbH, Dassel, Germany), blocked with5% skim milk in PBS for 30 minutes at room temperature, and then incubated at 4°C overnight with either anti-BVDV NS5A rabbit polyclonal antibody (produced in our laboratory using a recombinant BVDV NS5A as an immunogen) or anti-BVDV E2 mouse monoclonal antibody (15-C5, IDEXX Laboratories, Inc., Westbrook, Maine, USA) at a dilution of 1:1000 in PBS containing 1% skim milk. Following three washes with PBS containing 0.5% Tween 20, the membrane was incubated overnight at 4°C with for BVDV E2 or BVDV NS5A with diluted (1:3000) PBS containing 1% skim milk and either goat anti-mouse (IRDye 800CW, LI-COR Biosciences, Lincoln, NE, USA) or goat anti-rabbit (IRDye 800CW, LI-COR Biosciences, Lincoln, NE, USA), respectively. The specific protein band was visualized by using the Odyssey Imaging system and software (LI-COR Biosciences, Lincoln, NE, USA).

### Flow cytometry analysis

Flow cytometric analysis was performed to phenotypically characterize the harvested monocytes and Mo-DC, and to determine the effect of BVDV infection on Mo-DC cell surface marker expression. For monocyte characterization, three monoclonal antibodies (mAb) against MHCI (H58A), MHCII (H42A), and CD14 (MM61A) were used; while Mo-DC were characterized using six primary mouse mAb antibodies against bovine MHCI (H58A), MHCII (H42A), CD86 (IL-A190A), CD21 (BAQ15A), and CD14 (MM61A) (provided by VMRD Inc., Pullman, WA, USA), as well as CD205 (provided by Dr. Waithaka Mwangi, Texas A&M University, USA). To block the nonspecific binding, primary and secondary antibodies were diluted in 1% FBS in PBS. The primary antibodies were used as 1:100 dilution in 1% FBS in PBS while fluorescein isothiocyanate (FITC) labeled anti-mouse antibody (VMRD Inc., Pullman, WA, USA) was used as 1:1000 dilution in 1% FBS in PBS as secondary antibody. Mock-infected or BVDV- infected cells were collected at 0, 1,6,12, 24, 48 or 72 hour (s) post-infection. Collected cells were centrifuged at 200 g for 10 minutes at 4°C. Cell pellets were washed with 1%FBS in PBS. The cell staining was performed in round bottom 96 well plates. The collected cells were suspended in 50 μl primary antibody and incubated for 10 minutes at 4°C. The cells were washed two times with 1%FBS in PBS and suspended in 50 μl FITC labeled anti-mouse antibody. The cells were incubated in secondary antibodies for 10 minutes at 4°C and washed two times with 1% FBS in PBS. Following staining, the cells were suspended in 200 μl of 1% paraformaldehyde for fixing and analyzed using a dual-laser FACSCalibur (Becton-Dickson, Mountain View, CA, USA). To confirm nonspecific binding/ Fc receptor blockage, the cells were stained with FITC labeled anti-mouse antibody (secondary antibody) at each time points. Nonspecific binding of all primary antibodies was negligible based on these analyses. Each experiments was repeated a minimum of 3 times and significant different was determined by paired *T* test at 5% level of significance [44].

## Competing interests

The authors declare that they have no known competing interests.

## Authors’ contributions

Conception of the idea and design of the experiments are due in part to CCLC. The manuscript was written and drafted by CCLC, WM and MKSR. Flow cytometry analysis was performed with the guidance of AJY, MKSR, MFD, KP, and LJB conducted the research including blood collection, virus titration, Western Blot, and flow cytometry analysis. The bovine recombinant IL-4 and GM-CSF and guidance to generate Mo-DC were provided by WM. All authors read and approved the final manuscript.
